# Farmers’ indicators of soil health in the African highlands

**DOI:** 10.1016/j.catena.2021.105336

**Published:** 2021-08

**Authors:** Samuel Eze, Andrew J. Dougill, Steven A. Banwart, Susannah M. Sallu, Harriet E. Smith, Hemant G. Tripathi, Rashid N. Mgohele, Catherine J. Senkoro

**Affiliations:** a School of Earth and Environment, Faculty of Environment, University of Leeds, LS2 9JT Leeds, UK; b School of Biology, University of Leeds, LS2 9JT Leeds, UK; c Tanzanian Agricultural Research Institute (TARI), Mlingano Centre, Tanga, Tanzania

**Keywords:** Mountain soils, Soil health, Local soil knowledge, Sustainable land management, East Usambara Mountains

## Abstract

Improving soil health is necessary for increasing agricultural productivity and providing multiple ecosystem services. In the African Highlands (AH) where conversion of forests to cultivation on steep slopes is leading to soil degradation, sustainable land management practices are vital. Farmers’ awareness of soil health indicators (SHI) influences their choice of land management and needs to be better understood to improve communication between land managers and other stakeholders in agricultural systems. This study aims to collate and evaluate case study analyses of farmers’ awareness and use of soil health indicators in African Highlands. This is achieved by using a multi-method approach that combines a meta-summary analysis of AH’s SHI data from 24 published studies together with farmer interviews in the East Usambara Mountain region of Tanzania (EUM). Our findings show that farmers across the AH use observable attributes of the landscape as SHI. Out of 16 SHI reported by the farmers, vegetation performance/crop yield and soil colour were most frequently used across the AH. These were also the only two SHI that influenced farmers’ land management decisions in the EUM, where organic manure addition was the only land management option resulting from observed changes in SHI. Farmers’ use of only one or two SHI in land management decisions, as is the case in the EUM, seems to limit their choice and/or adoption of sustainable land management options, highlighting the need to increase awareness and use of more relevant SHI. This could be achieved by sharing SHI knowledge through learning alliances and agricultural extension service. Integration of farmers’ observation techniques and conventional soil testing in a hybrid approach is recommended for a more targeted assessment of soil health to inform appropriate and sustainable land management practices.

## Highlight

• African Highland farmers are aware of and use soil health indicators.
• The key indicators are vegetation performance and soil colour.
• Very few indicators influence land management decisions.
• There is a need for enhanced soil knowledge sharing across the highlands.
• Integration of local knowledge and conventional soil testing is needed.

## Graphical abstract

**Figure:**
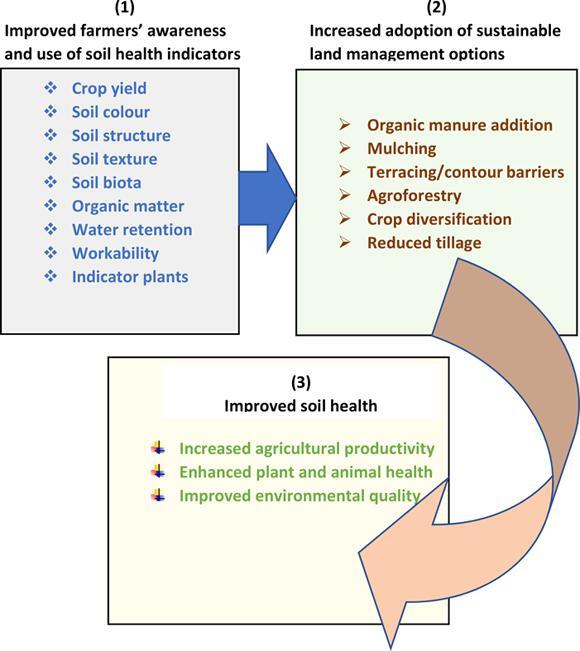

## 1 Introduction

Food insecurity remains a major challenge in sub-Saharan Africa (SSA), where a quarter of the population is estimated to be undernourished (FAO, 2015). The situation is compounded by high population density and poverty, unsustainable use of natural resources, climate change and extreme weather events, soil degradation, and low and declining agricultural productivity (Capitani et al., 2019). Marginal lands such as steep slopes in the African Highlands (AH) are intensively cultivated in an attempt to sustain the growing population, which exacerbates erosion and decline in soil health (FAO and ITPS, 2015). AH are defined as areas within the African continent that are 900 m or more above sea level, following Hamilton (1998) who showed that the soils, vegetation and climate above 850 m in the East Usambara Mountains (EUM) of Tanzania differed significantly from those below it and required different land management practices.

In many parts of the AH, such as the Matengo Highlands in Tanzania (Malley et al., 2006), Chipata Highlands in Zambia (Ajayi, 2007) and the eastern escarpment of the Great Rift Valley in Ethiopia (Karltun et al., 2013), farmers witness declining soil health, which they consider as one of the major constraints to agricultural productivity and household food security. The choice of land management practices is important as they influence soil health, defined as the continued capacity of the soil as a living system to provide multiple functions of sustaining biological productivity and health, and enhancing environmental quality (Doran and Zeiss, 2000). Climate-smart agriculture (CSA), an integrated landscape management approach that involves strategies to sustainably increase agricultural productivity, enhances resilience and adaptation to climate change and mitigate greenhouse gas emissions (Lipper et al., 2014), represents a set of approaches to improving soil health.

Despite concerns for soil health decline, farmers’ adoption of sustainable land management practices such as CSA remains low amongst smallholder farmers (Cordingley et al., 2015). Multiple factors have been linked to limited uptake of sustainable soil management practices, such as weak policy integration and limited institutional support (Chinseu et al., 2018), inadequate agricultural extension advice (Fisher et al., 2015), poor infrastructure (Kaweesa et al., 2018), farmers’ resource constraints (Kassie et al., 2015) and the neglect of farmers in the design of land management practices (Meijer et al., 2015). An additional important factor, often overlooked in discussions of underlying factors influencing land management decisions, is farmers’ understanding of soil health indicators.

Studies from Cameroon (Kome et al., 2018), Rwanda (Kuria et al., 2019), South Africa (Buthelezi-Dube et al., 2020) and Uganda (Pincus et al., 2018) show that highland farmers have good knowledge of soil health indicators. Farmers’ indicators of soil health such as soil colour, texture, consistency, moisture, organic matter, workability, structure, depth and temperature (Barrera-Bassols and Zinck, 2003) can enhance or hinder their adoption of recommended soil conservation measures, depending on whether they consider such measures to be beneficial in maintaining or changing the soil health. Certain soil attributes used by farmers as indicators of soil health, such as texture and effective soil depth, are not particularly sensitive to management changes, unlike attributes such as soil structure and organic matter content that can be modified via management activities (Barrios et al., 2006). Smallholder farmers tend to employ management practices that produce immediate benefits (Giller et al., 2009) as they often cannot afford the time lag or risks required to adopt longer-term management practices that have ongoing costs without delivering immediate benefits (Pannell et al., 2014). Focusing on the improvement of less sensitive soil health indicators can lead to frustration and abandonment of adopted practices when expected benefits appear not realised. In this case, adverse decisions for soil health can result from indicators that give false negative results, due to lack of sensitivity that does not show observable change even with incremental improvements in soil status. It is important to understand farmers’ use of indicators of soil health in order to effectively assess the impacts of land management practices and to improve mutual understanding between farmers, researchers, agricultural extension officers and local to regional land use planning officers.

Soil health indicators used by farmers across the AH have not yet been compiled, synthesized, nor critically examined to explore links to farmers’ land management decisions. There remains poor understanding of the most relevant SHI that influence farmers’ land management decisions, and uncertainty over whether commonly used SHI are sensitive to management changes and relevant for making timely management decisions for correcting soil health problems. An improved understanding of farmers’ SHI in AH is required to provide insights on some hidden knowledge-related factors affecting implementation and adoption of sustainable land management practices.

By combining a systematic literature review and farmer interviews from the EUM, this study contributes both empirical analysis from the Eastern Arc Mountains and a synthesis of farmers’ indicators of soil health across AH. The aim is to collate farmers’ indicators of soil health in the AH and to identify links to the implementation of sustainable land management practices. Specific objectives are to:

1. Compile soil health indicators used by communities across the AH;
2. Identify the most relevant soil health indicators used by farmers to make land management decisions in the AH;
3. Compare the most relevant soil health indicators used by farmers in the East Usambara Mountains with those used across the AH;
4. Assess the sensitivity of soil health indicators used by farmers in the AH to land management changes.

## 2 Methodology

2.1 Research approach

A multi-method approach was used, involving a combination of primary case study data from the EUM and a meta-analysis of secondary data across the AH. Interview data from the EUM case study helped to enrich the meta-analysis as farmers’ understanding of soil health indicators in that region of the AH has not previously been studied in this context. Similar methodological approaches, combining empirical and existing literature, have been used elsewhere, for example in environmental life cycle assessments (Huttunen et al., 2014) and sustainability assessments to support decision-making in agriculture (Marchand et al., 2014). Such a multi-method approach is fitting for this study, as it provides information on the breadth of farmers’ understanding of soil health across the AH and the complimentary case study context allows a more in-depth analysis of their understanding.

### 2.1 A case study from the East Usambara Mountains

**Fig. 1:**
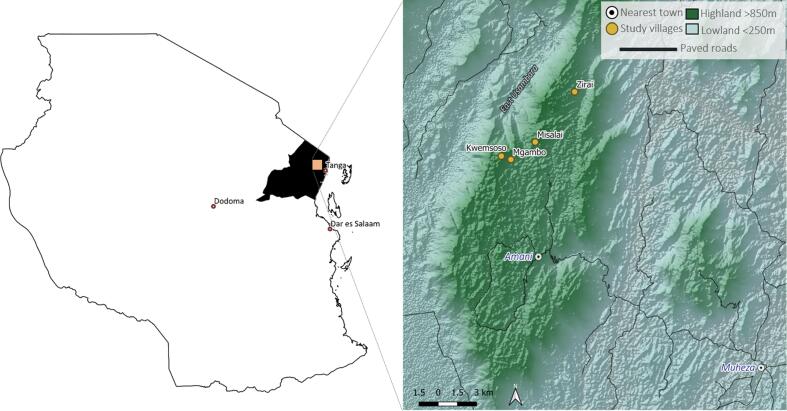
Tanzania showing the Tanga region and the villages where farmers were interviewed.

The EUM are located in the Tanga region of north-eastern Tanzania (Fig. 1), a separate block in the chain of Eastern Arc Mountains that stretches from Taita Hills in Kenya to Udzungwa Mountains in Tanzania. The EUM has steep slopes in the highlands of between 15 and 50%, mean annual rainfall of 1900 mm and mean annual temperature of 20 °C. The soils of the EUM are characterised by reddish brown to yellowish red Acrisols or Ferralsols with kaolinitic and sesquioxide clay minerals, and pH of 3.5–5.0 (NSS, 1989, Kirsten et al., 2016).

Communities in these mountains depend on high-biodiversity moist tropical montane forest ecosystems, which are under pressure from agricultural expansion, population growth, climate change and soil degradation (Kaihura et al., 1999, Reyes et al., 2005). Communities are interspersed between forest reserves and tea estates, and rely primarily on subsistence agriculture for their livelihoods with additional income from cash crop production and wage labour e.g. working in tea estates (Powell et al., 2011). Spices such as cardamom (*Elettaria cardamomum* (L.) Maton.), cinnamon (*Cinnamomum verum* J. Presl.), cloves (*Syzygium aromaticum* (L.) Merr.) and black pepper (*Piper nigrum* L.) are the main cash crops grown, mostly in subsistence agroforestry systems (Powell et al., 2013).

Sustainable and climate-smart land management practices on small parcels of land have been promoted in some villages in the EUM as part of an European Union’s Global Climate Change Alliance (GCCA + ) integrated adaptation programme (European Union, 2019). On-farm practices introduced through farmer-field schools have included: construction of *Fanya juu* terraces (trenches dug across slopes with excavated soils thrown uphill to form terraces), contour planting and use of grass strips to stabilise the soil and reduce erosion, agroforestry, addition of organic manure to improve fertility, diversification of crops to include perennial spices, and planting of drought tolerant varieties of maize.

A total of 50 semi-structured interviews were used to collect SHI data in the EUM between August and September 2019. Open-ended questions were conducted with 50 farmers from three villages in Muheza District, Tanga Region (Fig. 1). Interview questions were designed to understand farmers’ considerations of what healthy (good) soils are, and management decisions based on observed changes in soils. Interviews were conducted in Kiswahili on farmers’ fields, where soils could be observed and described by farmers. Participant responses were recorded in notebooks and translated into English by two Tanzanian researchers and data were subsequently digitised and processed in Microsoft Excel (2016 version).

### 2.2 Systematic literature review

To assess the soil health indicators used by farmer communities across the AH, a systematic literature review and an integrative meta-summary of findings based on the method described by Sandelowski and Barroso (2003) were conducted. The meta-summary approach uses quantitative logic in integrating qualitative research findings in such a way that the frequency of findings serves as a measure of validity.

Literature searches, restricted to the period between 1900 and April 24, 2020, were conducted via Google Scholar (GS) and Web of Science (WoS) databases for relevant scientific research articles. The search entry contained keywords relating to SHI and AH, that were combined using Boolean operators as follows: (“knowledge“ OR ”traditional knowledge“ OR ”indigenous soil knowledge“ OR ”farmer soil knowledge“ OR ”local soil knowledge“ OR ”folk soil knowledge“ OR ”native soil knowledge“ OR ”community soil knowledge“) AND (”soil health“ OR ”soil health indicator“ OR ”soil properties“ OR ”soil attributes“ OR ”soil fertility“ OR ”soil quality“ OR ”soil function“ OR ”soil productivity“) AND (”Africa*“ OR ”African mountains“ OR ”African uplands“ OR ”African highlands“ OR ”African montane“ OR ”African sub-montane“). The searches resulted in a total of 919 articles (226 from WoS and 693 from GS) with 897 articles retained for screening after 22 duplicates were removed.

Screening of articles was based on two inclusion criteria: (1) the study reported farmers’ indicators of soil health, soil quality, soil productivity or soil fertility; (2) the study was carried out in an AH. After title and abstract screening, 72 articles were retained and only 30 of these articles met the inclusion criteria following full text screening. Finally, a total of 24 articles (Tables A1 and A2) were reviewed after six articles were further excluded because they contained the same data reported in other included articles. Data on SHI and study locations were extracted from each of the articles and managed in Microsoft Excel (2016 version).

### 2.3 Data analysis

Empirical data collected from the EUM and review data were organised in spreadsheets and analysed separately. For consistency in data analysis, a technical analogue of farmers’ descriptions of soil attributes was used. For example, soil structure was used in place of “loose crumbly soil”. The frequency effect size of each soil health indicator was then calculated by dividing the number of studies that reported each indicator by the total number of studies from which data included in the *meta*-summary was extracted (Sandelowski and Barroso, 2003). This approach of effect size calculation based on frequency of studies rather than frequency of farmer reports was chosen because<50% of the studies had data on the number or percentage of farmers reporting SHI. For the EUM and country-specific analysis of frequency effect size, where only one study was found in that country, number of farmers that reported a particular indicator was divided by the total number of farmers interviewed. The percentage frequency effect size was used as a measure of the relevance of the SHI to the AH farmers and was divided into three categories: (1) major indicators (50–100%); (2) moderate indicators (20–50%); (3) minor indicators (0–19%).

The major, moderate and minor SHI were further grouped into permanent and modifiable indicators based on the classification system of Barrios et al. (2001). This was done to assess the sensitivity to land management of the SHI that are most relevant to farmers in the AH. A permanent indicator is one that is very difficult to change via land management activities whereas a modifiable indicator can be easily altered via management activities applied to the soil (Barrios et al., 2001).

## 3 Results

### 3.1 Indicators of soil health used by farmers across the AH

Findings from highlands in nine countries (Cameroon, Ethiopia, Kenya, Rwanda, South Africa, Tanzania, Uganda, Zambia and Zimbabwe) across central, eastern and southern Africa are reported. The majority of the 24 studies analysed were from five east African countries: Ethiopia (6), Kenya (4), Rwanda (4), Tanzania (3) and Uganda (3) (Table A3).

Farmers across the nine AH countries reported 16 parameters as indicators of soil health: soil colour, texture, structure, consistency, workability, water retention, drainage status, organic matter, fertilizer requirement, depth, degree of erosion, level of compaction, macro fauna population, vegetation performance/crop yield, weed type and the position on the slope where the soil is found (Table 1, Table 2, Table 3). Vegetation performance/crop yield and soil colour were the only two indicators of soil health reported by farmers in all the nine AH countries (Table A3). Farmers describe soil health indicators in various ways. For example, crop performance is described in terms of yield, health and vigour, growth and colour (Table 3).

**Table 1:** Soil health indicators used by farmers in the African Highland regions and their relevance groupings based on percentage frequency effect size and sensitivity to land management activities.

Soil health indicator	Percentage of total reviewed articles (n = 24)	Relevance of soil health indicator	Sensitivity property (permanent/modifiable – time needed in years)
Vegetation performance/crop yield	88	Major	<2 years
Soil colour	83		2–6 years
Soil texture	67		Permanent
Presence of weeds/indicator plants	63		2–6 years
Water retention	50		2–6 years
Workability/Ease of tillage	42	Moderate	>6 years
Soil depth	29		Permanent
Organic matter	25		2–6 years
Drainage	25		<2 years
Soil macrofauna	25		2–6 years
Soil structure	21		2–6 years
Erosion	17	Minor	>6 years
Slope position	17		Permanent
Compaction	17		<2 years
Fertilizer requirement	13		<2 years
Soil consistency	8		Permanent

**Table 2:** Soil health indicators (SHI) used by farmers in the East Usambara Mountains, their relevance groupings based on percentage frequency effect size and related management decisions.

Soil health indicator	Percentage of farmers (n = 50)	Relevance of soil health indicator	Management decisions based on observed changes in SHI
Soil colour	90	Major	Addition of farmyard manure in red soils to make it black
Vegetation performance/crop yield	32	Moderate	Addition of farmyard manure and incorporation of residues in soil to increase crop yield
Soil texture	32	Moderate	
Slope position	18	Minor	
Drainage	8	Minor	
Workability	4	Minor	
Presence of weeds/indicator plants	2	Minor

**Table 3:** Farmers’ descriptions of soil health indicators and their technical analogue.

Technical analog	Farmers’ descriptions
Vegetation performance/crop yield	Crop yield, crop quality, crop health and vigour, vegetation growth, vigorous plant growth, stunted growth, yellowing of crops, strong seedlings, darkish green crops, tall plants, large stalks,
Soil colour	Dark-coloured soils are good, red and white soils are bad, dark and black brownish soils are good, light soils are bad, black soils are good, dark grey soils are good,
Soil texture	Soft soil, coarse soils are bad, feel of the soil, soil is sandy, soil is dusty, soil is heavy
Presence of weeds/indicator plants	Presence of noxious weeds, presence of indicator plants, type of weed, type of invading plants and weeds, weed abundance, weed diversity, weeds that are easy to pull by hand, absence of fern-like weeds
Water retention	Not too wet and not too dry, contains water, holds water
Workability/Ease of tillage	Difficult to till, easy to plough
Organic matter	Soil lacks organic manure, soil have litter, soil have abundant crop residues
Drainage	Water drains the soil quickly, soil becomes too wet for long
Soil depth	Deep soils, shallow soils
Soil structure	Soil is crumbly, soil is loose, soil is clumped together
Soil macrofauna	Earthworms, Earthworm casts, beetle larvae, many worm holes
Slope position	Soil on hillside, soil on hill top, soil on hill bottom
Erosion	Surface soil is washed, rills and gullies in farms,
Compaction	Soil is compacted, soil stays loose
Fertilizer requirement	No fertilizer no yield, soil does not need fertilizer
Soil consistency	Hard, sticky, soil sticks to hoe and hand

### 3.2 The relevance of soil health indicators to farmers across the AH

The most frequently reported soil health indicators were vegetation performance/crop yield, soil colour, soil texture, the type of weeds growing and soil water retention. For example, the absence of specific weed species such as Bracken ferns and *Striga* spp., and the presence of *Trifolium decorum* chiov. and *Biden pilosa* L. were used by AH farmers as indicators of a healthy soil. Vegetation performance/crop yield and soil colour were the only parameters considered major soil health indicators in over 50% of the nine AH countries (Table A3).

Soil erosion, consistency, compaction and fertilizer requirement were the minor indicators of soil health across the AH. On a country-level, soil compaction was a major soil health indicator in Cameroon and Uganda; soil erosion and fertilizer requirement were also major indicators in Cameroon.

### 3.3 Sensitivity of farmers’ soil health indicators to land management

The 16 indicators of soil health reported by farmers across AH regions differ in their response to management activities. Four of the indicators i.e. soil texture, soil depth, slope position and soil consistency were classified as permanent indicators whereas the remaining indicators are modifiable via management activities (Table 1). Four out of the five most frequently reported soil health indicators in the AH regions are modifiable indicators. Vegetation performance/yield is modifiable within two years whereas soil colour, soil water retention and weed abundance are modifiable between two to six years.

### 3.4 Indicators of soil health used by farmers in the EUM and related management decisions

In the EUM, seven SHI were reported by farmers: soil colour, texture, workability, drainage status, vegetation performance/crop yield, weed type and slope (Table 2). Soil colour was the only reported major indicator of soil health - “*Udongo mwekundu*” (i.e. red soils) were perceived by farmers to be “bad” and less productive whereas “*Udongo mweusi*” (black or dark brown soils) were considered “good” and healthy. Vegetation performance/crop yield and soil texture were the moderate indicators and the remaining four parameters were minor indicators.

Out of the three most frequently reported SHI in the EUM, only soil colour and vegetation performance influenced management decisions. In red soils, farmyard manure was applied to make the soils black. According to one of the EUM farmers, “*In red soils, no manure, no yield*”. Similarly, poor crop performance led to the addition of farmyard manure and incorporation of crop residues in the soil. For example, “*I started adding manure because yield was declining”* was a common response from the EUM farmers.

## 4 Discussion

The results of both the systematic literature review and interviews from the EUM show that farmers in the AH use various attributes of the landscape as indicators of soil health. With the exception of our case study from the EUM, none of the studies included in the literature review was from any of the 13 Eastern Arc Mountain blocks in East Africa. This highlights the need for ethno-pedological research across this mountain chain and the importance of our case study in improving understanding of farmers’ awareness and use of soil health indicators in making land management decisions. Soil health is viewed by AH farmers in terms of the soil being “good” or “bad” for crop production. This is consistent with the concept of soil health in soil science literature where soil health is defined as “*the capacity of soil to function as a vital living system, within ecosystem and land-use boundaries, to sustain plant and animal productivity, maintain or enhance water and air quality, and promote plant and animal health*” (Doran and Zeiss, 2000). Although farmers often focus on only one component of the broader soil health concept, their indicators of soil health are very relevant in assessing the other components – environmental quality and animal health.

The 16 soil health indicators reported by AH farmers consist mainly of observable attributes of vegetation, soil and topography. These morphological descriptors are consistent with the results of previous studies (e.g. Barrera-Bassols and Zinck, 2003) in other regions of Africa and can be found in the global soil health indicator database (Jian et al., 2020). The soil health indicators reported by AH farmers also fit into five main sub-categories of soil health indicators (soil hydraulic properties, soil chemical fertility, soil aggregation, soil organic matter and biodiversity) identified by Velásquez et al. (2007), based on their provision of ecosystem services. Some of the indicators, particularly soil attributes such as structure, moisture retention, organic matter, and presence of fauna, underpin major ecosystem functions including carbon and nitrogen storage and nutrient transformations, greenhouse gas fluxes and water retention and transmission (Kibblewhite et al., 2007). Although all soil health indicators reported by farmers are relevant indicators that are also used in conventional soil health assessments (Bünemann et al., 2018, Jian et al., 2020), some of the indicators are more frequently used than others. The most frequently used indicators are those classified as major indicators that influence farmers’ assessment of soil health status and subsequent land management decisions (Bajgai and Sangchyoswat, 2018).

The EUM case study shows that vegetation performance/crop yield and soil colour were the only SHI influencing farmers’ land management decisions, with organic manure addition being the main management strategy used by farmers to address soil health-related problems particularly crop yield decline and when soil colour changes to red (a sign of declining soil fertility). Our review also shows that vegetation performance/crop yield and soil colour were the two most frequently used indicators across the AH regions, suggesting the importance of these SHI in farmers’ land management decisions in the region.

### 4.1 Vegetation performance/crop yield as a major soil health indicator

Farmers across the AH region described a healthy soil as one that supports good vegetation performance assessed in terms of having strong seedlings; tall, vigorous growth and darkish green colouration; large stalks and high crop yield. Vegetation performance is a good indicator of soil health that is sensitive to management changes, however, there are two main concerns that need to be considered. The first challenge associated with using vegetation performance as an indicator of soil health is that the performance indices are observed after planting and towards the end of the growing season when it is too late for corrective management activities such as fertilizer application to improve crop performance. Basing land management decisions on crop yield as an indicator of soil health will require a farmer to wait for at least a complete cropping cycle to be able to decide whether a soil is healthy or not. A second challenge is symptom misrepresentation (Barbedo, 2018), a situation where the plant growth characteristics described by farmers as indicators of unhealthy soil such as stunted growth and yellowing of leaves are a result of other factors such as pests and diseases. Comprehensive soil health assessment that reveals specific soil-related agricultural production constraints is required.

### 4.2 Soil colour as a major soil health indicator

Soil colour is the second major indicator of soil health across the AH, where farmers consider a healthy soil as one that is black, grey, brown, or dark-coloured whereas unhealthy soils are red, yellow, white or light-coloured. For example, some farmers in Ethiopia have used “getting red” to describe declining soil fertility (Karltun et al., 2013).

Soil colour reflects the predominant soil parent material in an area and the organic matter (OM) content. In the EUM, soil colour was the most frequently mentioned defining soil attribute used by 90% of interviewed farmers to assess soil health. The soils of the EUM are generally reddish brown to yellowish red in colour which reflects the heavily leached soils of the area with low activity clay minerals, kaolinites mixed with oxides and hydroxides of iron and aluminium (NSS, 1989). These reddish soils are acidic with low nutrient retention capacity (Kirsten et al., 2016), which aligns with farmers’ perceptions. Soil OM makes soils dark in colour (Spielvogel et al., 2004), thus the dark colour used by farmers to describe a “good” soil is an indication of OM content. The farmers’ understanding of colour as a key indicator of the productivity of their soils is consistent with scientific knowledge that the nutrient retention capacity of low-activity clay minerals is dependent on OM levels (Tan and Dowling, 1984).

In the southwestern Ethiopian highlands where Humic Nitisols with dark reddish brown colour predominate (Bezabih et al., 2016), a declining soil OM would make the soils become light in colour. This is consistent with the views of the farmers in this region that white soils are infertile (Bezabih et al., 2016). Findings therefore suggest that farmers in the AH region use soil colour as a proxy for soil OM. Farmers across the region also reported presence of organic materials in the soil as a moderate indicator of soil health and some believe that without the addition of organic manure the soils will remain unproductive (Asfaw and Ågren, 2007, Rushemuka et al., 2014). Some farmers in the AH (e.g. 90% of Girinka farmers in the highlands of Ngoma District, Rwanda and farmers in the EUM) are rightly focusing on OM management via the addition of organic manure as a means of improving soil health.

Considering the nature of the AH with steep slopes, it is important to be aware that addition of organic manure alone will not suffice in improving soil health due to erosion challenges. Support to farmers to employ erosion-control measures such as agroforestry and reduced tillage accompanied with terracing and contour barriers should be considered to minimize the loss of OM and nutrient-rich top soil (Wolka et al., 2018). African Highlands are a source of water to people living both in the highlands and downstream lowland areas. Controlling erosion will help mitigate downstream issues, such as sedimentation and eutrophication, leading to oxygen depletion, death of aquatic organisms and the overall decline in the water quality (Issaka and Ashraf, 2017).

The promotion of organic manure addition, agroforestry, construction of terraces and contour barriers in some villages in the EUM through the GCCA + project (European Union, 2019) is an important step towards improving soil health through soil fertility management and erosion control. These practices affect not only soil colour, the major SHI reported by farmers in the EUM case study, but other important SHI such as soil structure, soil macro-fauna, and soil chemical fertility. Though these were not reported to influence farmers’ land management decisions in the case study area. Farmers in some regions of the AH however, are aware of and use some SHI that are not being used by farmers in the EUM, thus highlighting the opportunity for knowledge sharing across the AH through, for example, learning alliances and agricultural extension services. Enhancing EUM farmers’ awareness and use of other relevant key SHI in addition to soil colour may help to better assess the impacts of sustainable land management practices that are being promoted in the region and enhance their adoption.

We acknowledge that, due to site specific differences, some SHI used in one context may not be suitable in another. However, in contexts where limited SHI influence land management decisions, such as the EUM, it may be useful to share knowledge on additional indicators, to support appropriate and sustainable land management.

### 4.3 Soil texture as a major soil health indicator

Findings from our literature review show that soil texture is the third most frequently used indicator of soil health in the AH. Soil texture is an important inherent soil property that influences many soil processes such as carbon fluxes and nutrient retention and availability (Silver et al., 2000), water retention and availability and root growth (Dexter, 2004). Some of these functions were also identified by the farmers. For example, farmers in western Cameroonian highlands believe that coarse-textured soils retain limited amount of water and nutrients and are infertile (Kome et al., 2018). Similarly, in the highlands of eastern Zimbabwe, farmers consider soils with high sand content to be degraded (Nezomba et al., 2015). Some of the farmers in both the EUM case study and across the AH region see water retention and drainage as well as workability (ease of tillage) as integral parts of soil texture whereas others consider these properties as separate but moderate soil health indicators.

Farmers’ views are consistent with scientific understanding of soil texture. However, it is important to note that soil texture does not change easily with management (Askari and Holden, 2015), creating challenges for farmers who desire immediate and observable changes. Instead, management practices that improve water and nutrient retention and release to plants are more appropriate, especially for coarse-textured (sandy) soils that farmers consider infertile (Kome et al., 2018). Practices such as retention of crop residues in the field and the addition of organic manures are key to improving not just soil colour but also other soil health indicators that are dependent on soil texture.

Using crop residues as livestock feed is considered by some researchers (e.g. Jaleta et al., 2013) as a major competitor to the retention of crop residues as mulch in mixed crop-livestock systems that characterize agricultural systems in SSA. For example, in the Emuhaya highlands of western Kenya, farmers prioritise the use of crop residues for feeding cattle, leading to low soil organic matter and nutrient content (Castellanos-Navarrete et al., 2015). In many crop-livestock systems in SSA, feeding livestock with crop residues takes precedence due to economic and cultural values of livestock (Giller et al., 2009). Further research is needed to understand the trade-offs between the multiple uses of crop residues and inform relevant policies.

### 4.4 Type of weeds/indicator plants as a major soil health indicator

The type of weeds growing in a field is also a major indicator of soil health used by some AH farmers. In the EUM case study, weed species were a minor indicator of soil health. The farmers in these highlands believe that healthy soils have higher weed diversity than unhealthy (poor) soils, and this is consistent with results of empirical studies (e.g. Santín-Montanyá et al., 2016). Weed species used by farmers as indicators of soil health differ across the AH regions. For example, Bracken ferns were used by farmers in the EUM and other weed species such as *Striga* spp., *Trifolium decorum* chiov. and *Biden pilosa* L. were used by farmers in other AH region as indicators of soil health. A common theme is that broad-leaf and succulent weeds characterise healthy soils whereas grasses dominate degraded (unhealthy) soils. *Striga* spp. are semi-parasitic plants that attach to and penetrate the roots of other plants, extracting nutrients from their hosts which enables them to thrive under poor soil health conditions (Kanampiu et al., 2018).

Weed type is an indicator of soil health that is modifiable in the medium term of two-six years (Table 1). Improving soil health will be reflected by the changes in weed abundance and diversity within a shorter period of time than some soil health indicators mentioned by the farmers, such as soil texture. As the response of weed species to management practices is contingent on site-specific conditions (Lee and Thierfelder, 2017), changing soil conditions due to specific management practices will affect weeds differently across the AH.

### 4.5 Less frequently used indicators of soil health in the AH

The soil health indicators used less frequently by farmers across the AH are soil consistency, compaction, erosion and fertilizer requirement. All these minor indicators except fertilizer requirement are related to other indicators that are more frequently used. For example, soil consistency is a function of soil texture and moisture content and relate to the soil’s workability (ease of tillage), which were more frequently used by the farmers. Similarly, drainage and ease of tillage were more frequently mentioned by the farmers than compaction.

Since the more frequently used (major and moderate) soil health indicators are related to the less frequently used (minor) ones, promoting the use of the minor indicators in the AH may not necessarily be worthwhile for extension services. In line with practice in soil health assessment, the use of a minimum set of key indicators helps to minimize cost of assessment and data redundancy (Askari and Holden, 2015). In the EUM case study where soil colour was the only major indicator of soil health, promoting the use of other major indicators used across the AH will help in making a more comprehensive soil health assessment and appropriate land management decisions.

Farmers use fertilizer requirement as a proxy for soil chemical fertility – a key element of soil health (Velásquez et al., 2007). In some parts of the AH (e.g. northern Ethiopia; Tesfahunegn et al., 2011), soils that require the addition of fertilizers for optimum crop production are considered by the farmers as unhealthy. Although this view is consistent with conventional understanding of soil fertility, assessing fertilizer requirement is challenging for resource-constrained farmers, which may explain its limited use across the AH and in the EUM. To ascertain specific soil nutrient deficiencies, quantitative assessments such as laboratory analysis are needed. Since scientific approaches are expensive and require technical knowledge, farmers instead rely on years of visual monitoring of crop responses to applied fertilisers, which may lead to a waste of resources.

Hybrid approaches to soil health assessment where farmers’ observational techniques are integrated with conventional soil testing (Barrios et al., 2006) are more appropriate. For example, conventional soil testing on areas identified by farmers can provide quantitative data to verify farmers’ assertions (Obour et al., 2020). It can also provide information on specific nutrient deficiencies and toxicities that can inform targeted fertilizer application or other nutrient management practices (Belachew and Abera, 2010). This will help in designing appropriate context-specific management practices and improving agricultural productivity.

## 5 Conclusion

Farmers across the African Highlands use observable landscape properties including attributes of soil, plant and topography as indicators of soil health, indicating that there is potential for increased involvement of farmers in the assessment of the impacts of land management practices on the agroecosystem. The farmers’ major indicators of soil health, except soil texture, can be easily modified via management activities and are most suitable for monitoring management-induced changes in the agroecosystem. When combined, the soil health indicators used by AH farmers are adequate for visual soil health assessments. However, the awareness and use of the indicators by the farmers differs from place to place, and only one or two indicators such as soil colour and vegetation performance influence land management decisions in different parts of the AH. This narrows the scope of land management options that farmers use for improving soil health. Knowledge-sharing across the AH (without disregarding the importance of local contexts) and promoting the use of appropriate soil health indicators through agricultural extension services can support assessment of soil health and better land management decisions. Farmers across the AH recognise the importance of soil chemical fertility as an important component of soil health, but they rely on morphological soil attributes due to the complex technical knowledge needed for quantitative soil testing. Hybrid approaches to soil health assessment that integrate farmers’ knowledge with scientific techniques will be essential to help improve soil health, agricultural productivity and wider ecosystem service benefits.

## Declaration of Competing Interest

The authors declare that they have no known competing financial interests or personal relationships that could have appeared to influence the work reported in this paper.
