# Supplementary material for: Farmers’ indicators of soil health in the African highlands
Source: Catena (Amst). 2021 Aug;203:105336. doi: 10.1016/j.catena.2021.105336 (PMC8191407; doi:10.1016/j.catena.2021.105336)
Supplement: Supplementary data 1 [file mmc1.docx]

**Supplementary information**

Table A1: African highland case studies reviewed and used for meta-summary analysis.

| Location | Elevation (m) | No. of farmers interviewed | Reference |
| --- | --- | --- | --- |
| Western Cameroon | 1600-2064 | 256 | Kome et al., 2018 |
| Southern Ethiopia | 1800-2250 | 110 | Asfaw and Ågren, 2007 |
| Southwestern Ethiopia | 501-3000 | 63 | Bezabih et al., 2016 |
| Ethiopia | 2100 | 100 | Karltun et al., 2013 |
| Northern Ethiopia | 2060-2650 | 42 | Tesfahunegn et al., 2011 |
| Southeast Ethiopia | 2200-2400 | 25 | Belachew and Abera, 2010 |
| Tigray, Ethiopia | 2000-2500 | 50 | Corbeels et al., 2000 |
| Central Kenya | 1500 | 60 | Mairura et al., 2007 |
| Kiambu, Kenya |  | 12 | Murage et al., 2000 |
| Western Kenya | 1140-1500 | 331 | Odendo et al., 2010 |
| Kitui, Kenya | 1000-1180 | 59 | Yageta et al., 2019 |
| Ngoma, Rwanda | 1400-1700 | 360 | Kim et al., 2013 |
| Western Rwanda | 1460-3000 | 150 | Kuria et al., 2019 |
| Southern Rwanda | 1700-2200 | 120 | Rushemuka et al., 2014 |
| Central southern Rwanda | 1550-1750 | 120 | Steiner, 1998 |
| Eastern South Africa | 945-1300 | 200 | Buthelezi‐Dube et al., 2020 |
| Tanzania |  | 1566 | Berazneva et al., 2018 |
| Mbinga, Southwestern Tanzania | 1200-1500 | 80 | Malley et al., 2006 |
| Gairo, Morogoro, Tanzania | 1000 | 60 | Masuki et al., 2009 |
| East Usambara Mountains, Tanzania | 904-1152 | 50 | This study |
| Southern Uganda |  | 20 | Brunner et al., 2008 |
| Kiboga, Central Uganda | 1100-1400 | 32 | Musinguzi et al., 2015 |
| Central Uganda |  | 48 | Pincus et al., 2018 |
| Eastern Zambia | 900-1200 | 302 | Ajayi, 2007 |
| Eastern Zimbabwe |  | 30 | Nezomba et al., 2015 |

Table A2: References of articles reviewed and used for meta-summary analysis.

| **References** |
| --- |
| Ajayi, O.C., 2007. User acceptability of sustainable soil fertility technologies: Lessons from farmers' knowledge, attitude and practice in southern Africa. *Journal of sustainable agriculture*, *30*(3), pp.21-40. |
| Asfaw, Z. and Ågren, G.I., 2007. Farmers’ local knowledge and topsoil properties of agroforestry practices in Sidama, Southern Ethiopia. *Agroforestry Systems*, *71*(1), pp.35-48. |
| Bezabih, B., Lemenih, M. and Regassa, A., 2016. Farmers perception on soil fertility status of small-scale farming system in southwestern Ethiopia. *Journal of Soil Science and Environmental Management*, *7*(9), pp.143-153. |
| Berazneva, J., McBride, L., Sheahan, M. and Güereña, D., 2018. Empirical assessment of subjective and objective soil fertility metrics in east Africa: Implications for researchers and policy makers. *World Development*, *105*, pp.367-382. |
| Brunner, A.C., Park, S.J., Ruecker, G.R. and Vlek, P.L.G., 2008. Erosion modelling approach to simulate the effect of land management options on soil loss by considering catenary soil development and farmers perception. *Land degradation & development*, *19*(6), pp.623-635. |
| Buthelezi‐Dube, N.N., Hughes, J.C., Muchaonyerwa, P., Caister, K.F. and Modi, A.T., 2020. Soil fertility assessment and management from the perspective of farmers in four villages of eastern South Africa. *Soil Use and Management*, 36, 250-260. |
| Karltun, E., Lemenih, M. and Tolera, M., 2013. COMPARING FARMERS'PERCEPTION OF SOIL FERTILITY CHANGE WITH SOIL PROPERTIES AND CROP PERFORMANCE IN BESEKU, ETHIOPIA. *Land Degradation & Development*, *24*(3), pp.228-235. |
| Kim, S.K., Tiessen, K.H., Beeche, A.A., Mukankurunziza, J. and Kamatari, A., 2013. Soil Fertility and Manure Management—Lessons from the Knowledge, Attitudes, and Practices of Girinka Farmers in the District of Ngoma, Rwanda. *Agroecology and sustainable food systems*, *37*(6), pp.631-658. |
| Kome, G.K., Enang, R.K. and Yerima, B.P.K., 2018. Knowledge and management of soil fertility by farmers in western Cameroon. *Geoderma regional*, *13*, pp.43-51. |
| Kuria, A.W., Barrios, E., Pagella, T., Muthuri, C.W., Mukuralinda, A. and Sinclair, F.L., 2019. Farmers' knowledge of soil quality indicators along a land degradation gradient in Rwanda. *Geoderma regional*, *16*, p.e00199. |
| Mairura, F.S., Mugendi, D.N., Mwanje, J.I., Ramisch, J.J., Mbugua, P.K. and Chianu, J.N., 2007. Integrating scientific and farmers' evaluation of soil quality indicators in Central Kenya. *Geoderma*, *139*(1-2), pp.134-143. |
| Malley, Z.J., Semoka, J.M.R., Kamasho, J.A. and Kabungo, C.V., 2006. Participatory assessment of soil degradation in the uplands of southwestern Tanzania: Implications for sustainable agriculture and rural livelihoods. *The International Journal of Sustainable Development and World Ecology*, *13*(3), pp.183-197. |
| Masuki, K.F.G., Mowo, J.G., Ley, G.J. and Mkavidanda, A.J.T., 2009. Farmers' knowledge in the management of soil and water in semi-arid Tanzania: opportunity for building farmers' resilience to climate change. |
| Murage, E.W., Karanja, N.K., Smithson, P.C. and Woomer, P.L., 2000. Diagnostic indicators of soil quality in productive and non-productive smallholders’ fields of Kenya’s Central Highlands. *Agriculture, Ecosystems & Environment*, *79*(1), pp.1-8. |
| Musinguzi, P., Ebanyat, P., Tenywa, J.S., Basamba, T.A., Tenywa, M.M. and Mubiru, D., 2015. Precision of farmerbased fertility ratings and soil organic carbon for crop production on a Ferralsol. |
| Nezomba, H., Mtambanengwe, F., Tittonell, P. and Mapfumo, P., 2015. Point of no return? Rehabilitating degraded soils for increased crop productivity on smallholder farms in eastern Zimbabwe. *Geoderma*, *239*, pp.143-155. |
| Odendo, M., Obare, G. and Salasya, B., 2010. Farmers' perceptions and knowledge of soil fertility degradation in two contrasting sites in western Kenya. *Land Degradation & Development*, *21*(6), pp.557-564. |
| Pincus, L., Ballard, H., Harris, E. and Scow, K., 2018. Seeing below the surface: making soil processes visible to Ugandan smallholder farmers through a constructivist and experiential extension approach. *Agriculture and Human Values*, *35*(2), pp.425-440. |
| Rushemuka, N.P., Bizoza, R.A., Mowo, J.G. and Bock, L., 2014. Farmers’ soil knowledge for effective participatory integrated watershed management in Rwanda: toward soil-specific fertility management and farmers’ judgmental fertilizer use. *Agriculture, ecosystems & environment*, *183*, pp.145-159. |
| Tesfahunegn, G.B., Tamene, L. and Vlek, P.L., 2011. A participatory soil quality assessment in Northern Ethiopia's Mai-Negus catchment. *Catena*, *86*(1), pp.1-13. |
| Yageta, Y., Osbahr, H., Morimoto, Y. and Clark, J., 2019. Comparing farmers' qualitative evaluation of soil fertility with quantitative soil fertility indicators in Kitui County, Kenya. *Geoderma*, *344*, pp.153-163. |
| Belachew, T. and Abera, Y., 2010. Assessment of soil fertility status with depth in wheat growing highlands of Southeast Ethiopia. *World Journal of Agricultural Sciences*, *6*(5), pp.525-531. |
| Corbeels, M., Shiferaw, A. and Haile, M., 2000. *Farmers' knowledge of soil fertility and local management strategies in Tigray, Ethiopia*. IIED-Drylands Programme. |
| Steiner, K.G., 1998. Using farmers' knowledge of soils in making research results more relevant to field practice: Experiences from Rwanda. *Agriculture, Ecosystems & Environment*, *69*(3), pp.191-200. |

Table A3: Soil health indicators used by farmers in the African Highland countries and their relevance based on frequency effect sizes from reviewed articles.

| Soil health indicator | Relevance of soil health indicator | | | | | | | | |
| --- | --- | --- | --- | --- | --- | --- | --- | --- | --- |
|  | Cameroon (n = 1) | Ethiopia (n = 6) | Kenya (n = 4) | Rwanda (n = 4) | South Africa (n = 1) | Tanzania (n = 3) | Uganda (n = 3) | Zambia (n = 1) | Zimbabwe (n = 1) |
| Vegetation performance /crop yield | Major | Major | Major | Major | Major | Major | Major | Major | Major |
| Soil colour | Major | Major | Major | Major | Moderate | Moderate | Major | Minor | Minor |
| Soil texture | Major | Major | Major | Major |  |  | Moderate | Minor | Minor |
| Presence of weeds /indicator plants | Moderate | Major | Major | Moderate | Major | Major |  | Minor | Major |
| Water retention | Minor | Moderate | Major | Moderate | Moderate | Moderate | Major |  | Minor |
| Workability/Ease of tillage | Moderate | Major | Major | Major | Moderate |  | Moderate |  |  |
| Organic matter | Major | Minor |  | Major | Moderate |  |  |  |  |
| Drainage |  | Minor |  | Major | Moderate |  | Moderate |  |  |
| Soil depth | Moderate | Moderate |  | Moderate |  | Moderate | Moderate |  | Major |
| Soil structure | Moderate |  |  | Moderate | Moderate | Moderate | Moderate |  | Major |
| Soil macrofauna |  | Minor | Major | Moderate | Moderate |  |  |  | Major |
| Slope position |  | Minor |  | Major |  |  |  |  |  |
| Erosion | Major | Moderate |  |  |  | Moderate |  |  |  |
| Compaction | Major | Minor |  |  |  |  | Major |  |  |
| Fertilizer requirement | Major | Minor | Minor |  |  | Moderate |  |  | Moderate |
| Soil consistency |  |  |  | Moderate | Moderate |  |  |  |  |
